# Effect of predictors on incidence rate of pregnancy among reproductive age women on antiretroviral therapy at public hospitals of Jigjiga and Harar Towns, Eastern Ethiopia: a retrospective cohort study

**DOI:** 10.1186/s12905-022-02135-9

**Published:** 2022-12-26

**Authors:** Abdi Wariyo, Lemessa Oljira, Wako Golicha, Gebisa Dirirsa

**Affiliations:** 1 School of Public Health, College of Health and Medical Sciences, Jigjiga University, Jigjiga, Ethiopia; 2 School of Public Health, College of Health and Medical Sciences, Haramaya University, Harar, Ethiopia; 3 School of Public Health, Bule Hora University, Bule Hora, Ethiopia; 4 Department of Environmental Health, College of Health and Medical Sciences, Haramaya University, P.O. Box 235, Harar, Ethiopia

**Keywords:** Antiretroviral therapy, Disease stage, Incidence rate, Pregnancy, Retrospective

## Abstract

**Background:**

Globally, Human Immunodeficiency Virus (HIV) is the leading cause of death in women of reproductive age and accountable for a quarter of deaths during pregnancy in sub-Saharan Africa including Ethiopia. Introduction of antiretroviral therapy to women living with HIV highly improves lifestyle and the desire to have children. A comprehensive understanding of baseline predictors of pregnancy among women receiving ART essential to reduces unintended pregnancies, appropriate care, and preventing transmission from mother to child.

**Objective:**

To determine the effect of baseline predictors on incidence rate of pregnancy among reproductive age women on antiretroviral therapy at public hospitals of Jigjiga and Harar town, Eastern Ethiopia from February 15 to march 15, 2020.

**Methods:**

Retrospective cohort study was conducted on randomly selected 420 HIV-infected women using data recorded from September 11, 2014, to September 10, 2019 in Jigjiga and Harar town in Eastern Ethiopia. Simple random sampling was used to select study subjects from each hospital. Data were entered to Epi data version 3.2 and exported to Stata version 14.2 for analysis. Kaplan–Meier failure, and Cox proportional hazards model were used to estimate the incidence, and to identify predictors of pregnancy respectively. Variables which were significant (*P* value < 0.05) in the multivariate analysis were considered independent predictors of pregnancy.

**Results:**

The overall incidence rate of pregnancy was 9.1 per 100 person-years (95% CI 7.19, 11.76). Being unadvanced HIV disease stage (AHR: 2.50; 95% CI 1.46, 4.19), having less than two children (AHR: 2.93; 95% CI 1.59, 5.40), and disclosed HIV status (AHR: 2.25; 95% CI 1.34, 3.79) were independent predictors of pregnancy.

**Conclusion:**

The incidence rate of pregnancy among reproductive age women on ART was found to be considerable. Being unadvanced HIV disease stage, having less than two children, and disclosed HIV status were independent predictors of pregnancy. Thus, tailoring counseling have to be designed to enhance better pregnancy planning and consecutive health outcomes.

**Supplementary Information:**

The online version contains supplementary material available at 10.1186/s12905-022-02135-9.

## Introduction

The Human Immunodeficiency Virus/Acquired Immunodeficiency Syndrome (HIV/AIDS) remains a serious public health challenge, as well as a social dilemma especially among women of reproductive age [1]. In 2018, around 18.8 million women of reproductive age globally, living with HIV [2]. Of these, 61% were residing in sub-Saharan Africa [2]. In Ethiopia, the prevalence of HIV/AIDS among women of reproductive age was 1.2% and the prevalence increases with increasing women’s age [3].

Introduction of antiretroviral therapy to women living with HIV highly improves lifestyle and the desire to have children [4]. Women who have HIV should seek treatment in accordance with WHO treatment guidelines because the virus can be passed on to their babies during pregnancy, childbirth, and breastfeeding [4]. Despite the fact that providing antiretroviral treatment to women living with HIV/AIDS has a tremendous effect on a mother's health and can virtually eliminate the risk of passing on the virus to a newborn, a birth from an HIV positive mother may result not only in an HIV positive baby but also in maternal death [5]. If proper care for the mothers is not in place, maternal death due to complications related to HIV and pregnancy can be very high [6]. Reports have shown that even in the presence of ART maternal mortality rates have been reported to be five times higher in HIV infected women than in uninfected women [7], and it is responsible for at least 20% of all deaths, a figure that is higher than any direct obstetric cause [8]. In Ethiopia, pregnancy related complications among women on ART are among the many health problems. Even if the country is constructing and equipping health facilities with staff and equipment, still morbidity and mortality due to complications related to pregnancy among women on ART are very high [9].

Evidence indicates that HIV-positive women continue to desire more children in the future, though to differing degrees in different contexts. According to studies from Canada and Tanzania, 69% and 37% of HIV-positive mothers want to have more children, respectively [10, 11]. Various studies in Ethiopia have found varying levels of fertility desire among HIV-positive women. According to research conducted in North Wollo [12], Addis Ababa [13], and Tigray region [14], the majority of HIV positive women desired to have more children.

Successful translation of HIV-prevention strategies requires a comprehensive understanding of the magnitude and baseline predictors of pregnancy among HIV positive women. This knowledge essential to reduce unintended pregnancies, appropriate care, and preventing transmission from mother to child. Therefore, this study aimed to determine effect of baseline predictors on incidence rate of pregnancy among reproductive age women receiving antiretroviral therapy at public hospitals of Jigjiga and Harar town, Eastern Ethiopia.

## Methods

### Study setting and period

This study was conducted at public hospitals of Jigjiga and Harar town, Eastern Ethiopia: Four hospitals, namely; Karamara general hospital, Sheikh Hassan Yabare Jigjiga university referral hospital (SHY-JURH), Hiwot Fana specialized university hospital, and Jugol hospital were included in the study conducted from February 15 to March 15, 2020.

Karamara general hospital and Sheikh Hassan Yabare Jigjiga university referral hospitals are located in Jigjiga town. Jigjiga, the administrative capital of the Somali region, is located 628 km east of Ethiopia's capital, Addis Ababa. Five governmental health institutions (3 health centers and 2 Hospitals) provide ART service for the catchment area population. Karamara general hospital and SHY-JURH are selected for the study. Karamara general hospital has been providing ART services for more than 17 years. As of 2020, 2907 and 179 HIV/AIDS patients have utilized ART services at the Karamara general hospital and SHY-JURH respectively, of whom 781 and 105 are reproductive age women, were on ART [15].

Hiwot Fana specialized Haramaya university hospital and Jugol hospitals are located in Harar town, 525 km from Ethiopia's capital, Addis Ababa. Hiwot Fana specialized Haramaya university hospital is the region's high load hospital, treating more than 50% of HIV patients. Both hospitals have been providing ART services for more than 18 years, and as of 2020, 1179 and 259 women of reproductive age are receiving ART at the two hospitals, respectively [15].

### Study design

Retrospective cohort study was used.

### Source population

The source population was all women on ART at public hospitals of Jigjiga and Harar town.

### Study population

The study population was all reproductive age women who received antiretroviral treatment at public hospitals of Jigjiga and Harar town, between September 11, 2014, to September 10, 2019.

## Inclusion and exclusion criteria

### Inclusion criteria

All reproductive age women, who were getting antiretroviral treatment in the hospitals of Jigjiga and Harar town, between September 11, 2014, to September 10, 2019 were included in the study.

### Exclusion criteria

Women who were getting pre-ART care and started ART based on option B+. Women with incomplete data on exposure and outcome were excluded from the study.

### Sample size determination

**Objective one:** The required sample size for objective one was determined by using formula for event, based on the following assumptions: the significance level of 5%, 80% power, hazard ratio = 2.05, and π1 and π2 are the proportions to be allocated to group1 and 2 and for equal allocation π1 = π2 = $\frac{1}{2}$. The incidence rate of pregnancy = 4.92 per 100 person years that converted to five years gives 0.246 person years in the study conducted in northwest Ethiopia [9].

- Event (Pregnancy) = 4(Z_α/2_ + Z_β)_^2^)/ (lnHR)^2^) = 60 [16]
- Where Z_α/2_ and Z_β_ are standard percentiles which give 1.96 (95% CI) and 0.84 (80%)
- n = Pregnancy/Pr (Pregnancy), Pr is the probability of Pregnancy and it is calculated by
- Pr (Pregnancy) = 1-(π1 S1(T) + π2 S2(T)) = 1-$\frac{1}{2}$ (S1(T) + S2(T)) where S(T) = exp(-λt), IR = λt
- and S1 (t5) = exp(-0.246) = 0.782, S2(t5) = exp(-0.246 *HR) = 0.604
- Pr (Pregnancy) = 1-(π1 S1 (T) + π2 S2 (T)) = 1- $$\frac{1}{2}$$ (0.782 + 0.604) = 0.307
- n = Pregnancy/Pr (Pregnancy) = $\frac{60}{0.307}$ = 195 (adding 10% for incomplete data) = 215

**Objective two:** Sample size for specific objective two was calculated using stpower Cox in Stata version 14.2 software packages. The estimation was based on assumptions with the power of 80%, α (type I) error of 5%, and the hazard ratio of 2.3 for educational status of patients with unable to read & write vs. formal education [9]. By considering, 10% for incomplete data during follow up period and in adjusting for censoring taking the overall probability of event 0.12 with 1:1 allocation ratio, and 0.5 of standard deviation. By having all the above parameters, the Stata command estimated sample sizes for the second specific objective was 420.

Finally, sample size for first and second objectives was compared, and the larger of two sample sizes was used to determine a minimum required sample size. Thus, the sample size for objective two 420 was selected as final sample size for the study.

### Sampling procedure

**Fig. 1:**
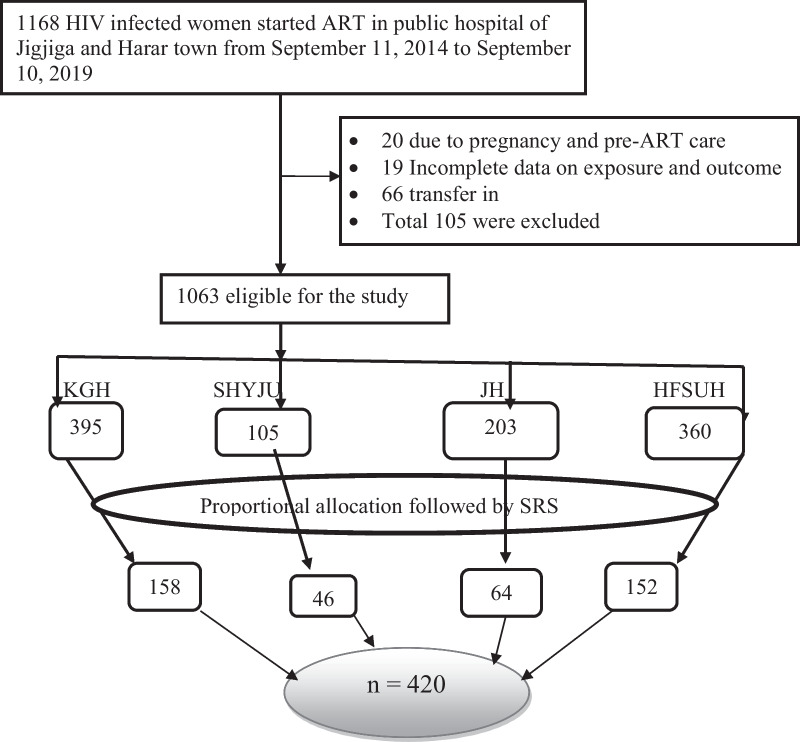
Schematic presentation of sampling procedure on the effect of predictors on incidence rate of pregnancy among reproductive age women on antiretroviral therapy at public hospitals of Jigjiga and Harar town, Eastern Ethiopia. KGH: Karamara general hospital; SRS: simple random sampling; SHYJURH: Sheikh Hassan Yabare Jigjiga university referral hospital; HFSUH: Hiwot Fana Specialized Haramaya university hospital; JH: Jugol hospital

Four public hospitals from Jigjiga and Harar city were included. Cards of 1168 women living with HIV from September 11, 2014, to September 10, 2019, was reviewed according to the date of ART initiation. Due to eligibility criteria, 105 patient cards were excluded, and 1063 patients were eligible for study. Eligible cards were from Karamara general hospital (37.6%), SHY-JURH (11%), Hiwot Fana specialized Haramaya university hospital (36.2%), and Jugol hospital (15.2%). Finally, each hospital's study subjects were selected using proportional allocation followed by simple random sampling (Fig. 1).

### Data collection tools and procedure

A structured data collection checklist was prepared in English to extract data from the records. Data was extracted from the hospital's main excel record. Data elements which were not included in the excel sheet were collected from ART intake forms and cards. The most recent laboratory results before starting ART were used as a baseline. If there were no pre-treatment laboratory tests, results obtained within one month of ART initiation were used as baseline. The data was extracted by eight nurses who work in ART clinics and supervised by two experienced BSc nurses. The patients̕ date of pregnancy was extracted from ART follow up registration form.

## Variables

### Dependent variable

Time to pregnancy in months.

#### Independent variables

Socio-demographic factors: Age, Place of residence, marital status, educational status, main occupation, number of children, and religion.

Clinical, laboratory, and treatment related factors: WHO clinical stages, CD4 count level, BMI, functional status, hemoglobin level, ARVs medication adherence, ART regimen given, opportunistic infection, cotrimoxazole prophylactic status, and disclosure of HIV status.

### Operational definitions

**Incidence of Pregnancy:** Incidence of pregnancy was considered as the first pregnancy after ART enrollment within the follow-up time [9].

**Time scale:** The time to event was calculated in months using the time between the dates of treatment initiation and the date of the event (pregnancy) or date of censoring. Thus, the minimum and the maximum follow up were 6 months and 60 months respectively.

**Censored:** If the exact time at which the event occurs is not observed, or if their last visit is any one of the following events; if lost to follow-up, transferred to another health facility, dead, and if not pregnant at the end of follow up [9].

**Lost to follow up:** A patient who has not attended the ART clinic for more than 90 days since the last scheduled appointment and whose whereabouts are unknown to the ART clinic staff [17].

**Transferred-out:** If the patients have moved to another health facility with confirmed written documentation of transfer out [17].

**Not pregnant:** If the patient does not experience the events at the end of the study.

**WHO clinical stages** (See Additional file 1).

**Advanced HIV disease stage:** Advanced HIV disease stage for adults is defined as having WHO clinical stage III or IV events [18].

**Unadvanced HIV disease stage:** Unadvanced HIV disease stage for adults is defined as having WHO clinical stage I or II events [18].

### Data quality control

Data quality was ensured during data collection, coding, entry, cleaning, and analysis. The data collectors and the supervisor were trained on the data collection procedures for two days and supervisor supervised the data collection process. The principal investigator oversaw the overall process. Completeness of data was checked in each day of activity and the necessary feedback was offered to data collectors the next morning. Moreover, double data entry was performed to prevent data entry error.

### Method of data analysis

Following the accomplishment of data collection activities, the data were entered to Epi data version 3.2 and exported to Stata version 14 for analysis. Mean with standard deviation, and median with inter quartile range for continuous variables, and frequencies with percentage for categorical variables were computed to describe a baseline characteristic of the participants. Incidence rate of pregnancy was determined from date of ART initiation to date of pregnancy recorded. The cumulative probability of pregnancy at every twelve months was estimated using Life table. Kaplan–Meier failure was used to estimate the probability of pregnancy after ART initiation. Crude hazard ratio test was carried out to identify candidate variables for the multivariable Cox regression model at *P* value < 0.25. Cox proportional hazards model was used to identify predictors of pregnancy and Variables which were significant (*P* value < 0.05) in the multivariate analysis were considered as independent predictors of pregnancy.

## Results

### Socio-demographic characteristics of the participants

A total of 420 HIV positive women from the record reviews were analyzed having a mean age of 31 (± 7 SD) years. About half; 232(55.2%) of the study subject were orthodox. About two-third of the study subject were urban by residence, less than two children by number of children with a magnitude of 329(78.3%), and 277(66.0%), respectively. Nearly half; of the study subjects were married and have no formal education with a magnitude of 192(45.7%), and 185(44.0%), respectively. About 155(36.9%) of study subjects were jobless (Table 1).

**Table 1:** Baseline socio demographic characteristic of the study participants-initiated ART in public hospitals of Jigjiga and Harar town, 2020, n = 420

Variable (n = 420)	Category	N (%)
Age	Mean ± S	31 ± 7
Religion	Muslim	131 (31.2%)
	Orthodox	232 (55.2%)
	Protestant	44 (10.5%)
	Other^a,b^	13 (3.1%)
Residence	Urban	329 (78.3%)
	Rural	91 (21.7%)
Marital status	Never married	77 (18.30%)
	Married	192 (45.7%)
	Widowed	72 (17.10%)
	Divorced	79 (18.80%)
Educational status	Illiterate	185 (44.0%)
	Primary	113 (26.9%)
	Secondary	80 (19.00%)
	Higher	42 (10.00%)
Occupation	Government employee	45 (10.70%)
	Private-employee	26 (6.200%)
	House wife	35 (8.300%)
	Daily laborer	97 (23.10%)
	No work	155 (36.9%)
	Merchant	38 (9.00%)
	Others^c^	24 (5.70%)
Number of children	≥ 2 children	143 (34.00%)
	< 2 children	277 (66.00%)

^a^Catholic

^b^Adventist

^c^Commercial sex workers

### Clinical, Laboratory, and Treatment related characteristics

In terms of baseline clinical and laboratory characteristics, about 90% of patients, 381 (90.7 percent), had begun ART with TDF + 3TC + EFV. About two-thirds of the study subjects were working by functional status, and good by ARVs medication adherence with a magnitude of 314(74.8%), and 333(79.3%), respectively. The median Hemoglobin level and CD4 cell counts of the study subjects was 11.5(4.5), and 223(258) respectively. Furthermore, the average body mass index (BMI) of the study subjects was 19.7 (3.6) kg/m2. Above half of the study subjects were yes by OIs and cotrimoxazole Prophylaxis status, and disclosed by HIV disclosure status with a magnitude of 238(56.7%), 244(58.1%), and 250(59.5%), respectively (Table 2).

**Table 2:** Baseline clinical, laboratory, and treatment related characteristic of the study participants-initiated ART in public hospitals of Jigjiga and Harar town, 2020, n = 420

Variable (n = 420)	Category	N (%)
BMI	Mean ± S	19.7 ± 3.6
Functional status	Working	314 (74.8%)
	Ambulatory	64 (15.20%)
	Bedridden	42 (10.00%)
Disease stage	Advanced	210 (50.0%)
	Unadvanced	210 (50.0%)
Hemoglobin	Median (IQR)	11.5 (4.5)
CD4 count level	Median (IQR)	223 (258)
OIs	Yes	238 (56.7%)
	No	182 (43.3%)
ART regimen	1c = AZT + 3TC + NVP	9 (2.10%)
	1d = AZT + 3TC + EFV	3 (0.70%)
	1e = TDF + 3TC + EFV	381 (90.7%)
	1f = TDF + 3TC + NVP	14 (3.3%)
	1j = TDF + 3TC + DTG	13 (3.1%)
Medication adherence	Good	333 (79.3%)
	Fair	47 (11.2%)
	Poor	40 (9.5%)
HIV serostatus disclosure	Disclosed	250 (59.5%)
	Not-disclosed	170 (40.5%)
Cotrimoxazole Prophylaxis	Yes	244 (58.1%)
	No	176 (41.9%)

### Incidence rate of pregnancy among HIV-infected women on ART care

**Fig. 2:**
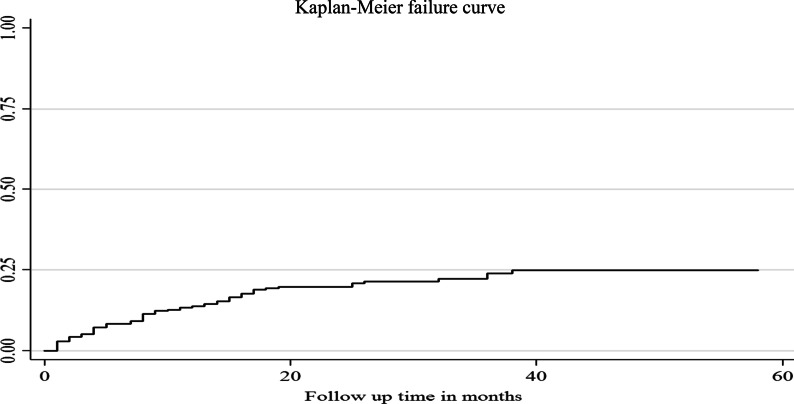
Kaplan–Meier failure curve estimate overall risk of having pregnancy among reproductive age women on ART at public hospital of Jigjiga and Harar town, 2020. From September 11, 2014 to September 10, 2019

In this study, there were 72 pregnancies, with the majority of them (69.4%) occurring in the first year of ART initiation. All of the study subjects (420) had contributed 9440 person month’s observations. The overall incidence rate of pregnancy was 9.1 per 100 person-years (95% CI 7.19, 11.76). The incidence rate of pregnancy in the entire cohort was 1.23, 0.59, 0.25, 0.33, and 0.000 per 100 person-year at 12, 24, 36, 48, and 60 months of ART initiation, respectively, indicating that as the year of follow up increases, the incidence rate of pregnancy decreases (Table 3). The Kaplan Meier failure curve depicts the probabilities of becoming pregnant at 20, 40, and 60 months of follow up, with the probability increasing over time (Fig. 2).

**Table 3:** Overall incidence rate of pregnancy for whole cohorts of reproductive age women on ART at public hospital of Jigjiga and Harar town, 2020. From September 11, 2014 to September 10, 2019

Cohort	Person-month	Failures	Rate	95% Conf. Interval
0–12	4060	50	0.0123	0.0094, 0.0164
12–24	2530	15	0.0059	0.0036, 0.0104
24–36	1586	4	0.0025	0.0009, 0.0090
36–48	906	3	0.0033	0.0010, 0.0162
> 48	358	0	0	
Total	9440	72	0.0076	0.0060, 0.0098

### Predictors of Pregnancy among HIV-infected women on ART care

In crude analysis; Age, Marital status, Level of education, Functional status, Number of children, BMI, Disease stage, Hemoglobin level, CD4 count level, OIs, ARVs medication adherence, Cotrimoxazole Prophylaxis, and disclosure of HIV status were variable candidate for multivariable Cox regression at *P* value < 0.25. Hence, place of residence was not included (Table 4).

**Table 4:** Baseline results of bivariable Cox regression analysis for predictors of pregnancy among reproductive age women on ART at public hospital of Jigjiga and Harar town, 2020

Variables	Categories	Follow up status		CHR (95%CI)	*P* value
		Pregnant (%)	Censored (%)		
Age	15–24	20 (23.3%)	66 (76.7%)	2.12 (1.07, 4.19)	0.032
	25–34	78 (18.1%)	172 (81.9%)	1.65 (0.89, 3.05)	0.109
	≥ 35	14 (11.3%)	110 (88.7%)	(1)	
Residence	Urban	55 (16.7%)	1274 (83.3%)	(1)	
	Rural	17 (18.7%)	74 (81.3%)	1.16 (0.67, 1.99)	0.600
Marital status	Never married	9 (11.7%)	68 (88.3%)	(1)	
	Married	42 (21.9%)	150 (78.1%)	2.05 (1.00, 4.27)	0.050
	Widowed	11 (15.3%)	61 (84.7%)	1.45 (0.60, 3.50)	0.407
	Divorced	10 (12.7%)	69 (87.3%)	1.12 (0.45, 2.75)	0.811
Level of education	No formal education	32 (17.3%)	3153 (82.7%)	0.72 (0.36, 1.48)	0.376
	Primary	19 (16.8%)	94 (83.2%)	0.75 (0.35, 1.62)	0.472
	Secondary	11 (13.8%)	69 (86.2%)	0.54 (0.23, 1.27)	0.159
	Certificate and above	10 (23.8%)	32 (76.2%)	(1)	
Number of Children’s	≥ 2 children	13 (9.1%)	130 (90.9%)	(1)	
	< 2 children	59 (21.3%)	218 (78.7%)	2.34 (1.28, 4.26)	0.006
Disease stage	Advanced	20 (9.5%)	190 (90.5%)	(1)	
	Unadvanced	52 (24.8%)	158 (75.2%)	2.43 (1.45, 4.07)	0.001
BMI	< 18.5 kg/m^2^	17 (10.7%)	142 (89.3%)	(1)	
	18.5-25 kg/m^2^	53 (22.7%)	181 (77.3%)	1.73 (1.00, 3.00)	0.049
	> 25 kg/m^2^	2 (7.4%)	25 (92.6%)	0.50 (0.11, 2.16)	0.351
Functional status	Unable to working	7 (6.6%)	99 (93.4%)	(1)	
	Able to Working	65 (20.7%)	249 (79.3%)	2.34 (1.07, 5.10)	0.034
Hemoglobin level	< 11	19 (9.5%)	181 (90.5%)	(1)	
	≥ 11	53 (24.1%)	167 (75.9%)	2.26 (1.33, 3.81)	0.002
CD4 count level in cells/mm^3^	< 200	19 (9.7%)	176 (90.3%)	(1)	
	200–350	16 (17.2%)	77 (82.8%)	1.57 (0.81, 3.06)	0.183
	> 350	37 (28.0%)	95 (72.0%)	2.62 (1.51, 4.56)	0.002
OIs	Yes	29 (12.2%)	209 (87.8%)	(1)	
	No	43 (23.6%)	139 (76.4%)	1.81 (1.13, 2.89)	0.014
ARVs medication adherence	Not adhered	4 (4.6%)	83 (95.4%)	(1)	
	Adhered	68 (20.4%)	265 (79.6%)	2.76 (1.00, 7.61)	0.050
Cotrimoxazole Prophylaxis	Yes	32 (13.1%)	212 (86.9%)	(1)	
	No	40 (22.7%)	136 (77.3%)	1.60 (1.00, 2.54)	0.049
HIV serostatus disclosure	Not-disclosed	21 (12.3%)	149 (87.7%)	(1)	
	Disclosed	51 (20.4%)	199 (79.6%)	1.69 (1.02, 2.82)	0.036

CHR, crude hazard ratio; CI, confidence interval

In multivariable Cox regression analysis, women with unadvanced HIV disease stage at baseline has increased risk of pregnancy by more than two-fold (AHR: 2.50; 95% CI 1.46, 4.19) compared to women with advanced HIV disease stage, after adjusting the number of children, and disclosure of HIV status. The risk of pregnancy was almost three (AHR: 2.93; 95% CI 1.59, 5.40) times higher among mothers who had less than two children compared to those who had two and above children. Women who had disclosed their HIV status was increase the risk of pregnancy by two folds (AHR: 2.25; 95% CI 1.34, 3.79) when compared to Women who had not disclosed their HIV status (Table 5).

**Table 5:** Results of the Multivariable Cox regression analysis for predictors of pregnancy among reproductive age women on ART at public hospital of Jigjiga and Harar town, 2020

Variables	Categories	Follow up status		CHR (95%CI)	AHR (95%CI)
		Pregnant (%)	Censored (%)		
Age	15–24	20 (23.3%)	66 (76.7%)	2.12 (1.07, 4.19)	1.46 (0.69, 3.07)
	25–34	78 (18.1%)	172 (81.9%)	1.65 (0.89, 3.05)	1.26 (0.67, 2.39)
	≥ 35	14 (11.3%)	110 (88.7%)	(1)	(1)
Number of children’s	≥ 2 children	13 (9.1%)	130 (90.9%)	(1)	(1)
	< 2 children	59 (21.3%)	218 (78.7%)	2.34 (1.28, 4.26)	2.93 (1.59, 5.40)**
Disease stage	Advanced	20 (9.5%)	190 (90.5%)	(1)	(1)
	Unadvanced	52 (24.8%)	158 (75.2%)	2.43 (1.45, 4.07)	2.50 (1.49, 4.19)**
Functional status	Unable to working	7 (6.6%)	99 (93.4%)	(1)	(1)
	Able to Working	65 (20.7%)	249 (79.3%)	2.34 (1.07, 5.10)	1.38 (0.58, 3.26)
Hemoglobin level	< 11	19 (9.5%)	181 (90.5%)	(1)	(1)
	≥ 11	53 (24.1%)	167 (75.9%)	2.26 (1.33, 3.81)	1.18 (0.48, 2.95)
CD4 count level in cells/mm^3^	< 200	19 (9.7%)	176 (90.3%)	(1)	(1)
	200–350	16 (17.2%)	77 (82.8%)	1.57 (0.81, 3.06)	0.68 (0.20, 2.37)
	> 350	37 (28.0%)	95 (72.0%)	2.62 (1.51, 4.56)	1.36 (0.34, 5.44)
OIs	Yes	29 (12.2%)	209 (87.8%)	(1)	(1)
	No	43 (23.6%)	39 (76.4%)	1.81 (1.13, 2.89)	0.72 (0.36, 1.43)
Disclosure of HIV Status	Not-disclosed	21 (12.3%)	149 (87.7%)	(1)	(1)
	Disclosed	51 (20.4%)	199 (79.6%)	1.69 (1.02, 2.82)	2.25 (1.34, 3.79)**

*P value < 0.05, **P value < 0.01, AHR: adjusted hazard ratio; CHR: crude hazard ratio, Bold symbol indicated variables significantly associated with the outcome variable

## Discussion

The overall incidence of pregnancy was 9.1 per 100 person-years. Being unadvanced HIV disease stage, having less than two children, and disclosed HIV status were independent predictors of pregnancy.

The incidence of pregnancy was 9.1 per 100 person-years in the entire follow up period. This finding is consistent with research conducted in western Uganda (90.7/1000 person-years) [19], Uganda (9.4/100 person-years) [20], and sub-Saharan Africa (9/100 person-years) [21].

This finding is lower than the studies done in Tanzania (12/100 person-years) [22], and Uganda (24.6/100 person-years) [23]. This disparity could be attributed to differences in access and quality of services, as well as differences in pregnancy diagnosis criteria. This result is also lower than that of a prospective population-based cohort study conducted in eastern Ethiopia [24], in which the incidence of pregnancy was 22.7/100 person-years. This difference could be explained by the fact that in Assefa et al.'s prospective cohort study, the study subjects were free HIV/AIDS people, whereas in this study, the study subjects were women with HIV/AIDS.

This finding is higher than that of studies conducted in London (13/1000 person-years) [25], Korea (3.57/100 person-years) [26], Canada (2.91/100 person-years) [27], Eight west African countries (2.9/100 person-years), Burkina Faso (5/100 person-years) [28], and studies from Ethiopia: Addis Ababa (6.5/100 person-years) [29], and Debre Markos (4.92/100 person-years) [9]. This difference may be due to differences in; inclusion and exclusion criteria, the size of records reviewed, the study period, and difference in baseline exposure to ART.

Being unadvanced HIV disease stage at the time of ART initiation has increased risk of pregnancy by more than two-fold, after controlling potential baseline confounders. This finding is in line with the findings of the studies conducted in eight west African countries and Ethiopia [29, 30]. Women with advanced HIV disease stage were less likely to be pregnant compared with unadvanced stage. This could be because being in advanced HIV disease stage leads to decreased general health and well-being, which may be associated with decreased sexual activity. Being unadvanced, on the other hand, implies good health and a lower risk of complications, which probably influences both the fertility and the fertility desire.

Women with fewer than two children were nearly three times more likely to become pregnant than women with two or more children. This finding is consistent with the findings of an Ethiopian studies [9, 29]. Women who had less than two children might have high fertility desire compared to those having two or more children. This might be due to the fact that, women who had two or more children more likely to utilize family planning services.

Women who had disclosed their HIV status had a twofold increase in the risk of pregnancy when compared to women who had not disclosed their HIV status. This finding is in line with the finding of a studies done in Uganda [20], and Ethiopia [31]. Communication between partners may play an important role in pre-conception planning behaviors. Additionally, disclosing an HIV-positive status to a sexual partner leads to safer sexual practices, social and emotional, and financial supports, which probably increase their fertility desire.

### Strength and limitations

This is one of the few studies in Ethiopia that explored effect of predictors on incidence rate of pregnancy among HIV infected women in ART era. Since this study was time to event analysis it enables us to consider contribution of censored study subjects. Due to a large number of missing data points, interesting variables such as family planning use, fertility desire, and intended and unintended pregnancies were not included. Thus, this finding should be interpreted with this limitation in mind.

## Conclusion and recommendation

The incidence of pregnancy among reproductive age women on ART is found to be considerable. Being unadvanced HIV disease stage, having fewer than two children, and disclosing HIV status were all independent predictors of pregnancy. Thus, tailoring counseling has to be designed to improve pregnancy planning and subsequent health outcomes. Beside this, health officers and data clerks working with recording HIV-positive patient’s data either in chart or database need training to improve quality of medical data especially in keeping dataset from missingness.

## Supplementary Information

**Additional file 1**. WHO clinical stage of HIV and detailed description of clinical stages.

## Data Availability

Almost all data are included in this study. However, additional data will be available from the corresponding author upon reasonable request.
