# Supplementary material for: Effect of predictors on incidence rate of pregnancy among reproductive age women on antiretroviral therapy at public hospitals of Jigjiga and Harar Towns, Eastern Ethiopia: a retrospective cohort study
Source: BMC Womens Health. 2022 Dec 26;22:548. doi: 10.1186/s12905-022-02135-9 (PMC9793559; doi:10.1186/s12905-022-02135-9)
Supplement: Supplementary file 1 — Additional file 1. WHO clinical stage of HIV and detailed description of clinical stages. [file 12905_2022_2135_MOESM1_ESM.pdf]

Supplementary Information's

Additional file 1: WHO clinical stage of HIV and detailed description of clinical stages.

**Clinical stage**	***Clinical description***	***Performance scale***
I	Asymptomatic<br>Persistent generalized lymphadenopathy	1. Normal activity
II	Weight loss <10%<br>Minor symptoms and infections	2. Normal activity
III	Weight loss, symptomatic<br>Diarrhea/fever > 1month	3. Bedridden>50% of days/month
IV	Symptomatic<br>AIDS-wasting syndrome<br>Severe opportunistic infections	4. Bedridden >50% of days/month

Detailed description of WHO clinical stages

Clinical stage 1: a person with confirmed HIV infection who is asymptomatic and/or persistent generalized lymphadenopathy (PGL)

Clinical stage 2: A person with confirmed HIV infection having:

- Moderate unexplained weight loss (<10% of presumed or measured body weight)
- Recurrent respiratory tract infections (sinusitis, bronchitis, otitis media, pharyngitis)
- Herpes zoster
- Angular cheilitis
- Recurrent oral ulceration
- Papular pruritic eruption
- Seborrhea dermatitis

- Fungal nail infections of fingers

Clinical stage 3: conditions where a presumptive diagnosis can be made based on a clinical sign or simple investigations:

- Severe weight loss (>10% of presumed or measured body weight)
- Unexplained chronic diarrhea for longer than one month
- Unexplained persistent fever (intermittent or constant for longer than 1 month)
- Oral candidiasis
- Oral hairy leukoplakia
- Pulmonary tuberculosis diagnosis in the last two years
- Severe presumed bacterial infections
- Acute necrotizing ulcerative stomatitis, gingivitis or periodontitis

Clinical stage 4: Conditions where a presumptive diagnosis can be made based on a clinical sign or simple investigations:

- HIV wasting syndrome
- Pneumocystis carinii pneumonia (PCP)
- Recurrent severe or radiological bacterial pneumonia
- Chronic herpes simplex infections
- Esophageal candidiasis
- Extrapulmonary TB
- Kaposi's sarcoma
- Central nerve system toxoplasmosis
- HIV encephalopathy
